# Gender Perspective of Risk Factors Associated with Disclosure of HIV Status, a Cross-Sectional Study in Soweto, South Africa

**DOI:** 10.1371/journal.pone.0095440

**Published:** 2014-04-17

**Authors:** Elisa Longinetti, Michele Santacatterina, Ziad El-Khatib

**Affiliations:** 1 Department of Public Health Sciences, Karolinska Institutet, Stockholm, Sweden; 2 Unit of Biostatistics – Institute of Environmental Medicine (IMM), Karolinska Institutet, Stockholm, Sweden; 3 Health Systems and Policy Research Group (Global Health), Department of Public Health Sciences, Karolinska Institutet, Stockholm, Sweden; 4 Global Health, Université du Québec en Abitibi-Témiscamingue, Rouyn-Noranda, Québec, Canada; University of Ottawa, Canada

## Abstract

**Background:**

Human Immunodeficiency Virus (HIV) status disclosure has been shown to provide several benefits, both at the individual and societal levels.

**Aim:**

To determine risk factors associated with disclosing HIV status among antiretroviral therapy (ART) recipients in South Africa.

**Setting:**

A cross-sectional study on risk factors for viremia and drug resistance took place at two outpatient HIV clinics in 2008, at a large hospital located in Soweto, South Africa.

**Methods:**

We conducted a secondary data analysis on socio-economic characteristics and HIV status disclosure to anyone, focusing on gender differences. Descriptive and multivariable logistic regression analyses were performed to model the associations between risk factors and HIV status disclosure. Additionally, descriptive analysis was conducted to describe gender differences of HIV status disclosure to partner, parents, parents in law, partner, child, family, employer, and other.

**Patients:**

A total of 883 patients were interviewed. The majority were women (73%) with median age of 39 years.

**Results:**

Employed patients were less likely to disclose than unemployed (odds ratio (OR) 0.36; 95% confidence interval (CI) 0.1–1.0; p = 0.05)). Women with higher income were more likely to disclose (OR 3.25; 95% CI 0.90–11.7; p = 0.07) than women with lower income, while men with higher income were less likely (OR 0.20; 95% CI 0.02–1.99; p = 0.17) than men with lower income. Men were more likely than women to disclose to their partner (p<0.01), and to partner and family (p<0.01), women were more likely than men to disclose to child and family (p<0.01), to child, family and others (p = 0.01).

**Conclusion:**

Being employed imposed a risk factor for HIV status disclosure, additionally we found an interaction effect of gender and income on disclosure. Interventions designed to reduce workplace discrimination and gender-sensitive interventions promoting disclosure are strongly recommended.

## Introduction

For women at the reproductive age, Human Immunodeficiency Virus (HIV) is the leading cause of death in sub-Saharan Africa (SSA) [1]. Young women account for 50–59% of people living with HIV (PLHIV) globally and in SSA [2]. The gender-related disproportion burden of the disease in this region has been increasing in the last 10 years and is due to both biological and cultural factors [3], [4]. There is an increased susceptibility to HIV due to violence against women caused by laceration during forced sex [5]. Also, women have limited access to preventive and treatment care, and tend to have low education level and income [5]–[8].

Both the Joint United Nations Programme on HIV and Acquired Immunodeficiency Syndrome (UNAIDS) and the World Health Organization (WHO) encourage voluntary disclosure of HIV status to bring beneficial results for the individual, family and sexual partners [9].

Beneficial results brought by HIV status disclosure include disclosure of HIV status to a sexual partner for prevention purposes [9]–[11], divulgence to health authorities for surveillance [9], and disclosure to family and health care workers for support and sharing of personal information [9], [10]. Finally, additional benefits gained by disclosure include an improvement in adherence to antiretroviral therapy (ART) and access to health care [12]–[14], as well as lower social related stigma [15]–[17].

Negative consequences of HIV status disclosure, such as discrimination [18], [24]–[26], are mostly faced by women, as they encounter possible violence and abandonment by friends and family [24], [27]. Women’s barriers to disclosure and consequent negative outcomes are due to their lower socio-economic status [8]–[10].

HIV status disclosure in South Africa is a topic that has been investigated with inconsistent findings, but only a few studies have been designed to identify gender differences [18]–[20]. Significant gender related differences in disclosure patterns were found in a literature review conducted by Mayfield et. al. [21] and in a cross-sectional study by Anglewicz et. al. [22] in Malawi.

However, a study conducted in Sweden by Asander et. al [24] among African families living in Stockholm, and other studies conducted in Louisiana, which focused on partner disclosure, rejected the hypothesis that gender is associated with HIV status disclosure [25]–[26]. In 2010, Deribe et.al. [27] found no significant gender difference in HIV status disclosure in Ethiopia. Nevertheless, contextual barriers such as education and marital status were unevenly distributed among men and women [27]. Key aspects of gender differences in disclosure of HIV status are still unclear and could be contributing factors to the gender related disproportion burden of disease [27].

Given the previous inconsistent findings about the relation between gender and HIV status disclosure, further studies are needed to properly capture this relationship. Based on women’s socio-economic barrier to disclosure [8]–[10] we hypothesized that women would be less likely to disclose their HIV status compared to men. This study aims to determine (1) what are the socio-economic factors associated with HIV status disclosure among men and women; (2) if there are any gender differences in patterns of HIV status disclosure among ART recipients in Soweto, South Africa. This enhanced understanding of the underlying factors and gender inequalities has the potential to improve the planning of gender-sensitive interventions by focusing on promoting disclosure, reducing the burden of disease, and diminishing additional related health costs.

## Methods

### Ethics Statement

The original study was approved by both (i) the research ethics committees at the University of Witwatersrand, Johannesburg, South Africa (M070721) and (ii) the Regional Medical Ethics Board in Stockholm, Sweden (Protocol 2008/3∶7) [28]. All participants signed written informed consent forms and were provided with a transportation reimbursement of 50 South African Rands (US$ 5) [28].

### Study Population

Secondary data analysis was performed on a cross-sectional study conducted at two outpatient HIV clinics in Soweto, South Africa [28]. The first is a non-governmental organization (NGO) research clinic and the second is a public clinic [28]. Both of them provided ART according to the South African National Antiretroviral Guidelines [29]. Participants were recruited between March and September 2008. Study participation was promoted using posters in clinics and pharmacy waiting areas. The inclusion criteria included: (i) being at least 18 years old, (ii) being on ART for at least 12 months and (iii) consent to be enrolled in the study.

Information was collected through structured interviews by trained nurses fluent in the three most widely-spoken languages in Soweto: English, Sesotho and isiZulu. After piloting, the interview questionnaire included 59 questions [28].

A total of 998 HIV patients were enrolled in the study and data were collected with respect to socio-demographic status and clinical characteristics including tuberculosis (TB) treatment, ART side-effects and adherence. Patients were grouped for antiretroviral line regimen and analysis of the present study was performed among first line regimen recipients (n = 833).

Study setting and selection criteria for participants are described in details in the original study report [28].

### Outcome Variable

HIV status disclosure was defined as the outcome of this study. HIV status disclosure was reported during structured interviews and defined as a positive answer to the question: “Have you disclosed your HIV status to other people?” In the case of a positive answer a succeeding question was asked: “If yes, who did you disclose your status to?” All the applicable answers were allowed: options were not mutually exclusive. Given the given the limited number of observations in some categories, we decided to collapse them in order to increase the number of observations as follows: (i) parents (ii) parents in law (iii) partner (iv) child (v) family (sibling+other family/household member) (vi) employer (vii) other (friend+other).

### Covariates

Information on gender was reported during structured interviews as well as information on age, education level, relationship status, employment status, total number of sources of income, income, type of housing and number of sexual partners. Information on type of clinic was collected through administrative records.

### Statistical Analysis

Initially, descriptive analysis was conducted to describe patients’ characteristics and patterns of HIV positive status disclosure. Chi-square and Fisher’s exact test (used when any expected frequency was less than 5) [30] was performed to determine whether gender is associated with a patient’s demographic and socio-economic characteristics and patterns of HIV status disclosure.

Secondly, odds ratios (ORs) were used to describe the strength of association between patients’ socio-economic risk factors, including education level, relationship status, employment status, number of sources of income, income level, type of housing and number of sexual partners and the binary outcome variable (i.e. HIV status disclosure, yes/no). Gender, age, and type of clinic were initially considered as potential confounders. The Mantel-Haenszel (M–H) method was used to estimate gender, age, and type of clinic-adjusted ORs. Comparisons between crude and adjusted estimations of ORs were conducted to investigate the possible significant confounding effect of gender, age, and type of clinic when studying the association between a patient’s socio-economic status as a risk factor and the outcome. A test of homogeneity was used to examine possible effect modifiers (data not shown) [30].

Finally, bivariate and multivariable logistic regression models [31] were performed to determine the presence of statistically significant associations between outcome and risk factors for which adjusted ORs and 95% confidence intervals (CIs) were calculated. Additionally, an interaction effect between gender and income was added into the model. An explanatory variable with a p-value ≤0.05 was considered as significant and contributed to the formulation of the final model.

Data analysis was performed using Stata Statistical Software: Release 12. (StataCorp. 2011. College Station, TX: StataCorp LP) [32].

## Results

### Sociodemographic and Clinical Characteristics of Study Participants

The study was based on 883 first line regimen recipients (646/883 women; 73%). Study population characteristics are summarized in Table 1 and Table 2 [28].

**Table 1:** Patient’s characteristics, demographics and socio-economic information (n = 883).

		All	By gender		
			Men	Women	P
		n (%)	n (%)	n (%)	
Age (median)	<39 years	432 (48.9)	91 (38.4)	341 (52.8)	**<0.01**
	≥39 years	452 (51.1)	146 (61.6)	305 (47.2)	
HIV status disclosedto anyone	No	31 (3.5)	6 (2.5)	25 (3.9)	0.33
	Yes	852 (96.5)	231 (97.5)	621 (96.1)	
Highest education level	No education or primary level	146 (16.4)	47 (19.8)	99 (15.3)	0.11
	Secondary or tertiary level	737 (83.5)	190 (80.2)	547 (84.7)	
Relationship status	Single, divorced, separated,widow	432 (49)	86 (36.3)	346 (53.7)	**<0.01**
	Married, sex partner,cohabitation	449 (51)	151 (63.7)	298 (46.3)	
Employment status	Not employed	589 (71.3)	135 (61.4)	445 (75)	**<0.01**
	Employed	233 (28.7)	85 (38.6)	148 (25)	
Number of sources of income	0	40 (4.6)	14 (6)	26 (4.1)	**<0.01**
	1	600 (68.6)	194 (82.5)	406 (63.4)	
	2	221 (25.3)	27 (11.5)	194 (30.3)	
	≥3	14 (1.6)	0 (0)	14 (2.2)	
Income	< median level of US$ 122	577 (65.4)	153 (64.6)	424 (65.4)	0.77
	≥ median level of US$ 122	306 (34.6)	84 (35.4)	222 (34.4)	
Type of housing	House	563 (63.8)	155 (65.4)	108 (63.3)	0.29
	Informal dwelling (shack)	212 (24)	48 (20.2)	164 (25.4)	
	Other (either flat, sharedroom or other)	107 (12.2)	34 (14.4)	73 (11.3)	
Type of clinic	NGO	431 (48.8)	106 (44.7)	325 (50.3)	0.14
	Public clinic	452 (51.2)	131 (55.3)	321 (49.7)	
Missed any pills during last weekend	No	833 (95.2)	221 (94)	612 (95.6)	0.33
	Yes	42 (4.8)	14 (6)	28 (4.4)	
Number of sexual partners last3 months (median = 1)	0	299 (33.9)	59 (25.1)	240 (37.3)	**<0.01**
	1	556 (63)	162 (68.9)	394 (61.2)	
	2–4 partners	24 (2.7)	14 (6)	10 (1.5)	
Knew HIV status beforestarting ART	0–12 months	487 (55.1)	147 (62)	340 (52.6)	**0.01**
	≥1 year	396 (44.9)	90 (38)	306 (47.4)	
Attendance to readiness program	No	12 (1.4)	2 (0.8)	10 (1.6)	0.15
	Yes	870 (98.6)	235 (99.2)	635 (98.4)	

**Table 2:** Patient’s characteristics, clinical information (n = 883).

		All	By gender		
			Men	Women	P
		n (%)	n (%)	n (%)	<0.01
CD4 cell count (median = 382 cells/µl)	≤200 cells/µl	111 (12.6)	49 (28.7)	62 (9.6)	
	>200 cells/µl	772 (87.4)	188 (79.3)	584 (90.4)	
Viral load (median = 399 HIV RNA copies/ml)	≤400 HIV RNA copies/ml	779 (88.4)	206 (87.3)	573 (88.8)	0.53
	>400 HIV RNA copies/ml	102 (11.6)	30 (12.7)	72 (11.2)	
Currently on TB therapy	No	869 (98.4)	231 (97.5)	638 (98.8)	0.17
	Yes	14 (1.6)	6 (2.5)	14 (1.6)	
Time on ART (median = 39 months)	12–23 months	154 (17.4)	47 (19.8)	107 (16.6)	0.33
	24–36 months	180 (20.4)	42 (17.7)	138 (21.4)	
	≥37 months	549 (62.2)	148 (62.5)	401 (62.1)	

The mean age for women and men was 39 (standard deviation (SD) 7.9) and 41 (SD 6.8), respectively. The majority (96.5%) disclosed their HIV status to at least one person, in addition they were evenly distributed among NGO (49%) and public (51%) clinics.

Overall, most of the patients had a secondary or tertiary education level (83.5%), one source of income (68.6%), and were unemployed (71.3%), where women were significantly more unemployed than men (75% vs. 61.4%; p<0.01). More than half of the participants (65.4%), had a median income lower than the equivalent of 122 US$ per month. Almost 64% lived in a house and reported having one sexual partner in the last three months.

Participants showed a complete adherence level to ART (only 4.8% reported missing any pills during the previous weekend), and the majority (98.6%) did attend a readiness program. Forty-five percent of patients knew about their HIV status over one year of their ART initiation.

More than half of the study participants (62.2%) had been on ART for longer than 37 months. Only 1.6% of the population were receiving TB therapy in conjunction with their ART.

The majority of patients (87.4%) had a CD4 cell count greater than 200 cells/µl, and 88.5% of them had a viral load (VL) lower than or equal to 400 HIV ribonucleic acid (RNA) copies/ml.

### Socio-Economic Factors Associated with HIV Status Disclosure

Table 3 shows socio-economic factors associated with HIV status disclosure. Consistent with results of the M–H test, the possible confounders including age, gender and type of clinic were not significantly associated with the outcome, therefore the final logistic regression models were not adjusted by age, gender or type of clinic. The estimate of the odds ratio for different levels of income show that the effect of gender on HIV disclosure seems to vary according to level of income. Women with higher income were more likely to self-report disclosure than women with lower income (OR 3.25; 95% CI 0.90–11.7; p = 0.07), while men with higher income were less likely to disclose than men with lower income (OR 0.20; 95% CI 0.02–1.99; p = 0.17).

**Table 3:** Socio-economic factors associated with HIV status disclosure: results from bivariate and multivariable logistic regression analysis (n = 883).

		HIV status disclosure		Crude OR	P	Adj.* OR	P
		No. n (%)	Yes. n (%)	OR (95% CI)		OR (95% CI)	
Highest education level	No education or primary level	7 (4.8)	139 (95.2)	1			
	Secondary or tertiary level	24 (3.3)	713 (96.7)	1.49(0.6–3.5)	0.36		
Relationship status	Single, divorced, separated, widow	17 (4.0)	415 (96.0)	1			
	Married, sex partner, cohabitation	13 (2.9)	436 (97.1)	1.37(0.7–2.9)	0.39		
Employment status	Not employed	13 (2.2)	567 (97.8)	1		1	**0.05**
	Employed	11 (4.7)	222 (95.3)	0.46(0.2–1.0)	0.06	0.36(0.1–1.0)	
Number of sources of income	0	1 (2.5)	39 (97.5)	1			
	1	20 (3.3)	580 (96.7)	0.74(0.1–5.7)	0.77		
	2	6 (2.7)	215 (97.3)	0.91(0.1–7.8)	0.93		
	≥3	3 (21.4)	11 (78.6)	0.09(0.0–0.9)	**0.05**		
Income	< median level of US$ 122	20 (3.5)	557 (96.5)	1		1	0.17
	≥ median level of US$ 122	11 (3.6)	295 (96.4)	0.96(0.4–2.0)	0.92	0.20(0.0–1.9)	
Type of housing	House	19 (3.4)	544 (96.6)	1			
	Informal dwelling (shack)	8 (3.8)	204 (96.2)	0.89(0.4–2.0)	0.78		
	Other (flat shared room or other)	4 (3.7)	103 (96.3)	0.90(0.3–2.7)	0.85		
Sexual partners last 3 months	0	14 (4.7)	285 (95.3)	1			
	1	15 (2.7)	541 (97.3)	1.77(0.8–3.7)	0.13		
	2–4 partners	2 (8.3)	22 (91.7)	0.54(0.1–2.5)	0.43		
Age (median)	<39 years	13 (3)	419 (97)	1			
	≥39 years	18 (4)	433 (96)	0.74(0.4–1.5)	0.43		
Gender	Men	6 (2.5)	231 (97.5)	1			0.09
	Women	25 (3.9)	621 (96.1)	0.64(0.3–2.0)	0.34	0.17(0.02–1.34)	
Type of clinic	NGO	16 (3.7)	415 (96.3)	1			
	Public clinic	15 (3.3)	437 (96.7)	1.12(0.5–2.3)	0.75		
Gender*Income						15.9(1.3–183.5)	**0.02**

*multivariable logistic regression model containing following risk factors: employment status, income, gender and interaction between gender and income.

Among study participants, employment status was significantly associated with HIV status disclosure (OR 0.36; 95% CI 0.1–1.0; p = 0.05). No significant association was present between HIV status disclosure and education level, relationship status, total number of sources of income, income, type of housing, or number of sexual partners. In addition, logistic regression analysis was stratified by gender, but no significant association was found (data not shown).

### Gender Perspective of Patterns of HIV Status Disclosure

Table 4 illustrates the rate of most common patterns of HIV status disclosure ranked by frequency. Multiple categories indicate combination patterns of disclosure.

**Table 4:** Rate of most common single and combination patterns of HIV status disclosure, ranked by frequency (n = 883).

	All	By gender		
		Men	Women	P
	n (%)	n (%)	n (%)	
Disclosure to parents and family	82 (9.6)	24 (10.1)	58 (8.9)	0.60
Disclosure to parents, partner and family	78 (9.2)	23 (9.7)	55 (8.5)	0.58
Disclosure to family member	62 (7.3)	20 (8.4)	42 (6.5)	0.31
Disclosure to parents, family and other	60 (7)	16 (7)	44 (7.1)	0.79
Disclosure to parents, partner, family and other	56(6.6)	20 (8.7)	36 (5.8)	0.18
Disclosure to partner and family	50 (5.9)	22 (9.3)	28 (4.3)	**<0.01**
Disclosure to family and other	45 (5.2)	13 (5.6)	32 (5.1)	0.68
Disclosure to partner	38 (4.5)	21 (8.9)	17 (2.6)	**<0.01**
Disclosure to child and family	37 (4.3)	3 (1.3)	34 (5.3)	**<0.01**
Disclosure to child, family and other	27 (3.2)	2 (0.9)	25 (4.0)	**0.01**

Approximately 10% of study participants chose to disclose their HIV status to parents and family, whereas only 9% disclosed to their partner in addition to parents and family. Around 7% informed each of parents, family members and others. Family members only and partner only were informed in 7.3% and 4.5% of the cases, respectively.

Disclosure to partner was significantly different among men and women, with men more likely to disclose exclusively to their partner (p<0.01) and to partner and family (p<0.01). Women were more likely to disclose their HIV status to child and family (p<0.01), to child, family and others (p = 0.01).

## Discussion

The present study focused on socio-economic factors associated with disclosure of HIV status and related gender differences among ART recipients in South Africa. Gender was not significantly associated with disclosure; however, the presence of an interaction factor of gender and income on disclosure was highlighted. Being employed has been identified as a risk factor for HIV status disclosure. Significant gender differences in patterns of HIV status disclosure were found; men were more likely to disclose to a partner only and to partner and family, whereas women were more likely to disclose to a larger family network including child and family, and child, family and others.

Participants showed a rate of HIV status disclosure (96.5%) higher than the average rate of disclosure in several countries from SSA, including Burkina Faso, Kenya, Rwanda, United Republic of Tanzania, ranging between 16.7% to 86% [33], [34]. Specific characteristics of our study population can explain a higher than average rate of HIV status disclosure. First, one inclusion criterion for the study consisted of being on ART for at least 12 months. Additionally, almost 45% of the participants knew about their HIV status for longer than 1 year before starting ART. The length of time of knowledge of serostatus in addition to availability and access to ART may have increased the likelihood of disclosure in our population. This hypothesis is consistent with recent evidence that illustrated increased disclosure of HIV status in SSA after the scale-up of ART [35].

The odds of disclosing status for women with high income were 3.25 times higher when compared to women with lower income (see Table 3). Women with low socioeconomic status tend to avoid disclosure of HIV status due to fear of rejection and violence [6]–[8]. Higher income and higher socio-economic status seems to reduce fear of abandonment by a partner and family, leading to increased rates of disclosure. The odds of disclosure for men with a high income were 80% less likely when compared to men with lower income (see Table 3). Men with higher income might be less willing to disclose their HIV status due stigma and discrimination issues in the workplace [21].

Employment status seemed to be a risk factor for disclosure of HIV status, with employed people 69% less likely to disclose their HIV status than unemployed people. Mayfield et al. [21] conducted a literature review on disclosure of HIV status among heterosexuals adults living with HIV, focusing on disclosure within workplace. Stigma and discrimination in the workplace were identified as possible causes for the low rate of disclosure found in that setting [21]. Previous research has suggested that employers don’t have the appropriate knowledge about HIV, ART and its success in turning the HIV epidemic into a manageable, chronic-like disease [21], [36]. This lack of education leads to discrimination and inappropriate employment terminations [21], [36]. Employers should therefore be the target of pilot educational programs focused on disseminating knowledge of PLHIV. Interventions to address workplace stigma should also be implemented.

Although both genders reported disclosure to their partner, relationship status did not seem to be associated with HIV status disclosure. Our findings contrast with previous research that shows being in a relationship is positively associated with disclosure [10], [33], [35], [37]–[40].

Income, total number of sources of income and type of housing were considered as proxies of socio-economic status, yet they were not associated with disclosure of HIV status in our study. Instead, higher socio-economic status has been previously shown to lead to an increased rate of disclosure [33], [35], [38]. The length of time of knowledge of serostatus might have diluted the effect of socio-economic status on disclosure towards a null association.

In contrast with previous research that described an increased risk of not disclosing HIV status for uneducated people [33], [39], [41], [42], education level did not seem to have an effect on disclosure of HIV status. Also, number of sexual partners did not seem to be associated with disclosure.

Contrary to expectations, age, gender and type of clinic did not have a confounding effect on the relationship between HIV status disclosure and the exposure variables (employment, education level, relationship status, total number of sources of income, income, type of housing, or number of sexual partners).

The most frequent pattern of disclosure was to both parents and family (9.6%), followed by disclosure to parents, family and partner (9.2%) without significant gender differences. Disclosure to a family member (7.3%) is the most common disclosure pattern when disclosure is made to only one category, and a family member is always included in the most frequent patterns of HIV status disclosure, except when disclosure is made only to the partner (4.5%). Mills et al. [43] showed that individuals on ART who disclose to family members are more likely to demonstrate a good adherence to treatment, consistent with this finding only 4.8% of study participants reported missing any pills during the previous weekend. Given the cross-sectional nature of this study, it is not possible to assess a temporal relationship between adherence to ART and disclosure to family members, but our research seems to confirm the association.

Disclosure to a sexual partner has been shown to be predicted by an advanced HIV status stage [33], [35], [38] and associated with increased CD4 cell count [44]. Disclosure to a partner only (4.5%) was an uncommon pattern of disclosure in our study population, probably due to the early disease stage of many participants. Advance stage of HIV disease has been defined as having a CD4 cell count below 200 cell/µl [45], and in our study, 87.4% of individuals had a CD cell count >200 cell/µl.

Men were more likely to disclose exclusively to their partners than women. Similarly, it has been suggested that women are less likely to disclose to their partner for fear of abandonment [22], and men prefer to confide to their spouse [46]. In contrast numerous studies found no association between gender and disclosure to partner [24]–[26].

Women were more likely to disclose to child and family, to child, family and others. Sowell et al. [47] also reported African women trust relatives more in disclosing HIV status.

Overall, when individuals chose to disclose their HIV status, they were inclined to communicate it to more than one individual, as previously reported in the literature [21]. Length of time since diagnosis may have impacted the number of confidants informed [21], [48].

### Strengths and Limitations

This study presents several strengths. First, it is one of the few studies specifically designed to investigate gender differences in disclosure of HIV status in SSA [27]. We studied gender perspective and contributed new results to the controversial findings obtained previously.

To our knowledge, this is the first study to indicate an interaction effect of gender and income on HIV status disclosure; hence it’s not possible to compare the varied effect of gender on HIV disclosure according to income level to other studies.

Most previous studies focused exclusively on disclosure with respect to one category, either a partner or family member. In our study, gender differences in patterns of HIV status disclosure were thoroughly studied. When asking “Who did you disclose your status to?,” all the applicable responses were allowed. The data obtained was retained in the analyses and by studying all the permutations and combinations of HIV status disclosure no information was lost.

A major limitation of this study was the disproportion between number of individuals that disclosed their HIV status and number of individuals that did not (853 vs. 31 respectively). Given the rarity of the non-disclosure event small-sample bias might have affected the maximum likelihood estimation of the logistic model [49]. Overall lack of power impeded stratification by gender for the logistic regression models.

The study was conducted among ART recipients attending an NGO and a public clinic, therefore disclosure of HIV status might have been overestimated and results might not be generalizable to other settings of PLHIV.

Participants of the study volunteered to be part of the research, and might have presented different characteristics from the general population. However it has been estimated that between 10% and 20% of eligible patients volunteered to be part of the study [28]. Even though the sample population was not selected randomly, demographic characteristics were consistent with a study conducted by Rosen et al. [50] on a large number of sites in South Africa [28].

Information on exposure was self-reported and might have been affected by recall bias. More specifically non-differential misclassification of total number of sources of income might have led to a dilution of its effect on disclosure. Since data was collected through structured interviews, social desirability bias might have also occurred. For instance, people might have underreported the number of sexual partners or modified other sensitive information such as income and sources of income. However, volunteer participation in the study should have minimized the risk of social desirability bias.

The cross-sectional nature of the study invokes methodological limitations. First, it was not possible to differentiate between causation and association, given the lack of information on temporal relationships between some of the exposure variables and HIV status disclosure. Employment status and relationship status might have negatively changed after disclosure. Additionally, complete interpretations of the findings cannot be extrapolated from the cross-sectional study itself, but can only be hypothesized and studied more thoroughly by way of cohort studies.

## Conclusion

To conclude, this study suggests that being employed imposes fear in PLHIV with regard to disclosing their HIV status; reduced willingness to disclose might be related to stigma and discrimination in the workplace. Efforts to educate employers about PLHIV and interventions designed to reduce workplace discrimination are strongly recommended. We also suggest future studies focusing on the interaction effect found between gender and income on HIV status disclosure.

Given the gender differences found in patterns of disclosure, planning of future interventions should focus on promoting disclosure to the partner among women and to family members among men. These findings have significant public health implications: targeted gender-sensitive interventions have the potential to increase overall disclosure, thereby reducing the stigma and discrimination associated with the HIV epidemic.
